# *Xenopus tropicalis*: Joining the Armada in the Fight Against Blood Cancer

**DOI:** 10.3389/fphys.2019.00048

**Published:** 2019-02-01

**Authors:** Dionysia Dimitrakopoulou, Dieter Tulkens, Pieter Van Vlierberghe, Kris Vleminckx

**Affiliations:** ^1^Department of Biomedical Molecular Biology, Ghent University, Ghent, Belgium; ^2^Cancer Research Institute Ghent (CRIG), Ghent, Belgium; ^3^Department of Biomolecular Medicine, Ghent University, Ghent, Belgium

**Keywords:** *Xenopus*, CRISPR/Cas9, leukemia, T-ALL, genome editing, thymus, tumor suppressor genes, cancer

## Abstract

Aquatic vertebrate organisms such as zebrafish have been used for over a decade to model different types of human cancer, including hematologic malignancies. However, the introduction of gene editing techniques such as CRISPR/Cas9 and TALEN, have now opened the road for other organisms featuring large externally developing embryos that are easily accessible. Thanks to its unique diploid genome that shows a high degree of synteny to the human, combined with its relatively short live cycle, *Xenopus tropicalis* has now emerged as an additional powerful aquatic model for studying human disease genes. Genome editing techniques are very simple and extremely efficient, permitting the fast and cheap generation of genetic models for human disease. Mosaic disruption of tumor suppressor genes allows the generation of highly penetrant and low latency cancer models. While models for solid human tumors have been recently generated, genetic models for hematologic malignancies are currently lacking for *Xenopus.* Here we describe our experimental pipeline, based on mosaic genome editing by CRISPR/Cas9, to generate innovative and high-performing leukemia models in *X. tropicalis*. These add to the existing models in zebrafish and will extend the experimental platform available in aquatic vertebrate organisms to contribute to the field of hematologic malignancies. This will extend our knowledge in the etiology of this cancer and assist the identification of molecular targets for therapeutic intervention.

## Introduction

*Xenopus laevis* and *Xenopus tropicalis* have been used extensively in developmental and cell biology, biochemistry, functional genomics and immunology. Embryos develop externally and tadpoles are transparent, features that facilitate experimental manipulation and post-factum analysis of animals (Robert and Ohta, 2009; Banach and Robert, 2017). Interestingly, in contrast to *X. laevis* and teleosts, *X. tropicalis* has a true diploid genome with high conservation of gene synteny with the human genome (Hellsten et al., 2010), making it an attractive biomedical genetic model organism. The immune system of *Xenopus* demonstrates striking similarities to that of mammals and a considerable number of immunological studies have already been performed (Banach and Robert, 2017). Recent advances in genome editing technologies have now made it possible to efficiently disrupt gene function in *Xenopus*, reinforcing it as an organism for modeling human disease, including the generation of solid tumor models (Van Nieuwenhuysen et al., 2015; Naert et al., 2016). Evidently, these genome editing strategies could also be applied for detailed studies in immunology and especially in generating models for hematologic malignancies. Here, we elaborate on strategies to generate and analyze hematologic malignancy models in *X. tropicalis*. We discuss how these *Xenopus* models can be applied for identifying driver and modifier genes and cancer dependency factors with possible therapeutic potential.

## The Immune System of *Xenopus Tropicalis*

The innate immune system of *Xenopus* consists of the same cell types as mammals and their primary function is to eradicate infected cells. Neutrophils, basophils, eosinophils as well as polymorphonuclear cells, macrophages dendritic cells and lymphocytes have all been detected (Hadji-Azimi et al., 1987; Robert and Ohta, 2009; Neely et al., 2018). Toll like receptor (TLR) genes are present and execute the same role as their mammalian counterparts in recognizing pathogen-associated molecular patterns (PAMPs; Roach et al., 2005). *Xenopus* has NK cells that eliminate virally infected and cancer cells by cytotoxic activity (Horton et al., 2000; Horton et al., 2003). Furthermore, innate immunity includes the three activation pathways of the complement system (classical, alternative and lectin) as well as antimicrobial peptides secreted by the epidermal layers (Zasloff, 2002).

Also the overall pattern and function of adaptive immunity is conserved with mammals. Orthologs of all major mammalian immune genes have been identified (Schwager et al., 1988, 1991; Chretien et al., 1997; Shi and Ishizuya-Oka, 1997; Flajnik, 2018). IgM is widely expressed and, similar to IgX (homolog of mammalian *IgA*), its expression is not dependent on the thymus (Tochinai, 1976). In contrast, expression of IgY (homolog of IgG and IgE) is thymus dependent and is detected in the thymus, spleen and peripheral blood (Mussmann et al., 1998). Next to those three major Ig isotypes, IgD has been reported (Ohta et al., 2006; Flajnik, 2018). Accessory molecules like the B7 family (and B7 receptor) (Hansen et al., 2009), cytokines like the TNFSF family (and TNFSF receptor) (Bernard et al., 2007), IFN gamma and the interleukins IL-2, -6, -7, -17, -21 and -23 have all been identified (Qi and Nie, 2008). B cell receptors (BCR) recognize specific epitopes on foreign antigens and T-cell receptors (TCRs) recognize short peptides presented by MHC class I and II. CD4 and CD8 T cells recognize and bind to MHC class II and MHC class I epitopes, respectively. Naive T cells demand an extra stimulatory signal (by costimulatory molecules B7 and CD40) to execute an immunological response. Activated T cells propagate and differentiate into cytotoxic T lymphocytes and CD4 T helper cells. The latter release cytokines that act either on CD8 T cells and B cells, or directly on pathogens. After immunological response, the majority of T cells die through apoptosis, except for T memory cells, which orchestrate secondary immune responses.

Despite the similarities to mammals, there are some fundamental developmental differences that confer special features to the *Xenopus* immune system. (1) The fetal liver is the main site for hematopoiesis, unlike the bone marrow (BM) in mammals (Ciau-Uitz et al., 2014). In adult *Xenopus*, the BM functions as a reservoir pool (Robert and Ohta, 2009), while no BM is present in tadpoles (Du Pasquier et al., 2000). (2) Lymph nodes are absent in *Xenopus*, and the spleen functions as a major secondary lymphoid organ, which interestingly is deprived of germinal centers (GCs; Du Pasquier and Schwager, 1991; Du Pasquier et al., 2000; Neely et al., 2018). *Xenopus* also lack follicular dendritic cells but their function in the spleen is taken over by “double-duty” antigen presenting cells (Neely et al., 2018). (3) During metamorphosis, the thymus undergoes histolysis and 50–90% of thymic lymphocytes die (Du Pasquier and Weiss, 1973; Du Pasquier et al., 1989). The thymic remnants shift toward the tympanum and tissue is regenerated simultaneously with a second wave of lymphoid stem cells colonizing the renewed thymus (Bechtold et al., 1992; Robert and Ohta, 2009). Premature B cells diminish during metamorphosis and regenerate in the liver at Nieuwkoop stage 60. In adult life, the major site of B cell differentiation shifts from the liver to the spleen (Hadji-Azimi et al., 1987). The larval type BCR repertoire is less variable compared to the adult. This could be explained by deficiency of N-nucleotide diversity during BCR rearrangement (Du Pasquier et al., 2000). However, VH diversity is similar between larvae and adults (Du Pasquier et al., 2000). Even though the *Xenopus* spleen has no GCs, somatic hypermutation occurs in adults and larvae. As in mammals, αβ T cell differentiation is thymus dependent (Robert and Ohta, 2009). Little information is available regarding the TCR repertoire. However, the TCR gene rearrangements (both larvae and adult stage) exhibit many similarities to mammals (Robert et al., 2001).

Because of its unique and conserved features, *Xenopus* provided important insights concerning regulation of MHC expression and immunotolerance. MHC class expression differs between larvae and adult and thus affects differently the tolerance toward skin grafts (Du Pasquier and Flajnik, 1990; Rollins-Smith et al., 1997). Moreover, *Xenopus* is a reliable system for immune-cancer studies. *Xenopus* can develop lymphoid tumors from which cell lines have been derived (Du Pasquier and Robert, 1992; Robert et al., 1994). These cell lines have been obtained from different strains and exhibited differential tumorigenicity, which was attributed to divergent expression of MHC Ia and MHC Ib genes (Goyos et al., 2007).

## Hematologic Malignancies

Leukemia is among the leading cancers worldwide (Bray et al., 2018). In adult patients, the most frequent types are acute myeloid leukemia (AML) and chronic myeloid leukemia (CML). Acute lymphoblastic leukemia (ALL), a malignant transformation and propagation of lymphoid progenitor cells in the BM, peripheral blood and various extramedullary sites, affects both adults and children (Van Vlierberghe and Ferrando, 2012; Terwilliger and Abdul-Hay, 2017). Most incidences of ALL are derived from the B cell lineage (B-ALL) while 25% of adult ALL and 15% of pediatric ALLs originate in the T cell lineage (T-ALL; You et al., 2015). B-ALL involves characteristic chromosomal translocations such as *t*(12;21) [*ETV6-RUNX1*], *t*(1;19) [*TCF3-PBX1*], *t*(9;22) [*BCR-ABL1*] and rearrangements of the MLL locus (Woo et al., 2014). T-ALL most frequent mutational events include activation of the Notch pathway, deregulation of cell cycle regulators and mutations in chromatin remodeling complexes (Van Vlierberghe and Ferrando, 2012). Although prognosis for B-ALL and T-ALL in general is good, still 15–20% of patients experience disease relapse (Cooper and Brown, 2015; You et al., 2015). In addition, patients often experience severe therapy related toxicities (Van Vlierberghe and Ferrando, 2012). Thus, identification of more effective and less toxic therapies is important. More effective therapies are also required for AML, where older patients, and those who exhibit relapsed disease, demonstrate poor prognosis (Dombret and Gardin, 2016). For CML patients treated with targeted therapies, like tyrosine kinase inhibitors, prognosis is significantly improved (Jabbour and Kantarjian, 2018). Animal models have already contributed majorly to leukemia research and have expanded the molecular biomedical knowledge, allowing the design, development and testing of novel anti-leukemic drugs.

## Genetic *Xenopus Tropicalis* Models for All

The majority of mouse ALL models have been generated by transgenic strategies or by cells transduced retrovirally to express mutant proteins or genomic aberrations (chromosomal translocations, activating and inactivating mutations) associated with ALL (Gutierrez et al., 2014; Jacoby et al., 2014; Kohnken et al., 2017). In addition, xenografts models of human ALL samples engrafted in immunocompromised mice have also been generated. Xenografts models enlighten topics that were previously difficult to address concerning ALL microenvironment, leukemic stem cells, prognostic markers and new therapeutic agents (Gopalakrishnapillai et al., 2016). The last decade has also seen the emergence of leukemia models in zebrafish, mostly mediated by mosaic transgenic expression of oncogenes such as MYC in the lymphocytic lineage. However, details regarding the established murine and zebrafish ALL models go beyond the scope of this perspective.

As mentioned before, the recent progress in genome editing technologies has created a true revolution in generating models for human disease. This also applies to *X.* *tropicalis*, which unlike zebrafish (Taylor et al., 2001; Howe et al., 2013) favorably has a true diploid genome (Naert et al., 2017). Evidently, homology between human and mice is greater compared to *X. tropicalis*. Nevertheless, signaling pathways that are involved in TCR and BCR formation as well as immune system cell types are highly conserved (Robert and Ohta, 2009). Therefore, *X. tropicalis* is suitable for modeling hematologic diseases. Targeting of tumor suppressor genes (TSGs) by CRISPR/Cas9 (or TALENs) results in F_0_ mosaic mutants, commonly mentioned as crispants. In these crispants, the cell clones that acquired bi-allelic loss of function (LOF) of the TSG may be able to form tumors.

**FIGURE 1:**
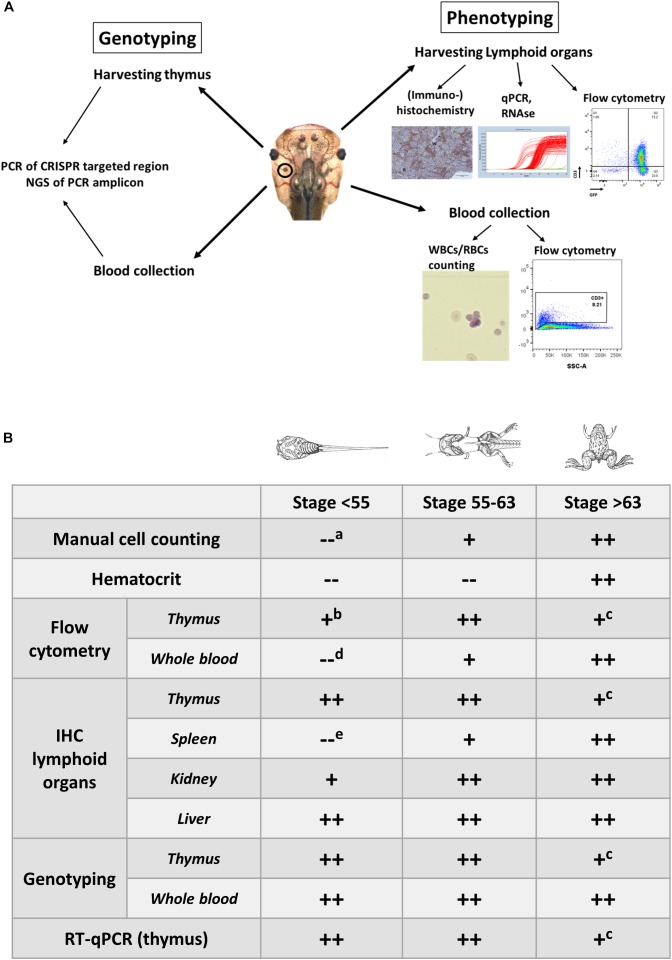
**(A)** Overview illustrating the experimental approaches to document the development of hematologic malignancies. **(A)** Scheme documenting the different types of analysis performed to investigate the presence of hematologic malignancies. Genotyping is performed on the blood and the dissected thymus by PCR amplification of the CRISPR/Cas9 targeted regions followed by deep sequencing of the PCR fragments and analysis of the INDEL signatures (left). Phenotyping is done by manual counting of the blood cells or by flow cytometry. In addition, lymphoid organs such as the thymus, and spleen are subjected to immunohistological analysis and transcriptomic profiling. Other organs like the kidneys and the liver are evaluated for the presence of proliferating and disseminating lymphoblasts (right). **(B)** Timing of the analyses that can be performed for assessing the presence of leukemic disease and to evaluate disease progression. Legend: “++,” “+” and “–” refer to straightforward, difficult and impossible to nearly impossible to perform, respectively. Analysis can be impossible to do due to (a) immature cells jeopardizing cell discrimination, (b) too low input of thymocytes for flow cytometry in early stage tadpoles, (c) shrinking of thymi in older animals, which impedes successful dissection, (d) aberrant scattering in immature cells, (e) extremely small size of the spleen in early stage tadpoles, which therefore is difficult to dissect. Drawings adopted from Xenbase (http://www.xenbase.org/anatomy/alldev.do).

Our lab published two genetically engineered tumor models in *X. tropicalis* (Naert et al., 2017). In both cases, solid tumors were obtained (e.g., desmoid tumors and retinoblastoma) and histological and immuno-histochemical evaluation, as well as genotyping, was straightforward and further confirmed these tumors to closely resemble their human counterparts (Naert et al., 2017). Recently, we also generated leukemia models in *Xenopus* by inducing LOF mutations in TSGs that are frequently mutated in T-ALL. While genotypic and phenotypic analysis of solid tumor models in *X. tropicalis* is straightforward, this is not the case for hematologic malignancies. In mice and humans, characterization of hematologic malignancies relies primarily on immunophenotyping with sets of antibodies recognizing specific surface markers on the transformed leukocytes. Unfortunately, such antibodies are largely lacking for *Xenopus*. Therefore, we designed and applied alternative strategies inspired by those used to analyze solid tumors. Our strategies, encompass both phenotypic and genotypic analysis of F_0_ mosaic mutants that are symptomatic leukemic (Figure 1).

### Phenotypic Analysis of F_0_ Crispants

Embryos are injected in the blastomeres that contribute to the definitive blood and animals are raised until they present symptoms of leukemic disease such as abnormal swimming behavior, lethargy, pallor and hemorrhagic spots. Animals with these symptoms are sedated and whole blood is withdrawn from the heart by glass capillaries. Identification of leukocytes in the blood is complicated by the fact that amphibians, like fish, birds and reptiles, have nucleated erythrocytes (Maxham et al., 2016). Unfortunately, red blood cell lysis buffer, usually applied to lyse erythrocytes and facilitate leukocytes isolation, cannot be used. Hence, blood from tadpoles is processed either by manual cell count in a hemocytometer or by flow cytometry.

#### Analyzing the Blood (Figure 1)

A first indication for leukemia may be abnormal hematocrit levels. However, this analysis is not possible for tadpoles and young froglets since it requires larger amounts of blood (∼50 μl). For manual cell counting, whole blood is diluted in Natt-Herrick solution (Maxham et al., 2016). By using a common hemocytometer, single blood cells are counted and categorized as white blood cells (WBCs), red blood cells (RBCs) or platelets, to calculate the WBC/RBC ratio. Natt-Herrick staining allows discrimination between myeloid derivatives and lymphocytes. Wright-Giemsa staining of a blood smear can also be used as an alternative way to identify overrepresentation of lymphoblast-like cells. In our experience, analysis of blood smears can be complicated by partially lysed erythrocytes.

Compared to manual cell counting, flow cytometric analysis is more quantitative, especially if the blood sample can be stained for lymphocyte markers. Even though the markers available for *Xenopus* are very limited, we are still able to define whether the leukemia has a T cell or B cell origin. For now, no good myeloid markers are available. For larval stage blood (or for metamorphic stages) we suggest staining for CD8 and CD3. Both are used as T cell specific surface markers. However, cytoplasmic CD3, detected upon cell permeabilization, is a marker of immature lymphoblast, which are clonally expanded in T-ALL (Robert and Ohta, 2009; Van Vlierberghe and Ferrando, 2012). Furthermore, larval and post-metamorphosis stage blood can be stained for MHC class II and cytoplasmic IgM, markers for B cells (Loftin et al., 1985). Next to these early lymphocyte markers, IgY staining can be included for identifying more mature B cells (Robert and Ohta, 2009). While the current lymphocyte surface and cytoplasmic markers allow us to discriminate between T- and B-ALL, they do not allow to define the different ALL subtypes. Of note, a recent report describes the use of acridine orange staining to discriminate different blood cell types by flow cytometry in *Xenopus* (Sato et al., 2018).

Analogs to zebrafish, a transgenic *rag2:EGFP* line in *X*. *tropicalis* can reinforce and facilitate phenotypic analysis of leukemic animals. Since *rag2* guides development and maturation of both T and B cells (Langenau et al., 2003), both cell populations will be GFP positive in these transgenic animals. Performing CRISPR/Cas9 injections in this transgenic line to generate ALL F_0_ mosaic crispants, will offer unique opportunities. Firstly, due to the transparency of larval stages, circulating and infiltrating lymphoblasts can be detected. Secondly, GFP positive lymphocytes/lymphoblasts can be easily separated by flow cytometry. This obviates the need for lymphocyte markers. Thirdly, sorted lymphoblasts can be transplanted into immunocompromised animals to evaluate the tumorigenic potential of these leukemic cells/blasts. Importantly, all aforementioned applications have already been used successfully in zebrafish (Langenau et al., 2003, 2005). In our lab, we have established a r*ag2:EGFP* F_0_ line and a *rag2*^+/-^ lines for these experiments. These tools will strengthen the validity of our model and facilitate future applications, especially for compound screening.

#### Analyzing Internal Lymphoid Organs (Figure 1)

Next to the blood, also the analysis of the lymphoid organs like the thymus, spleen and liver can be informative regarding leukemia establishment and progression. Healthy spleens, contain white and red pulp, that are well delineated by a boundary layer (Manning, 1991; Lametschwandtner et al., 2016). The red pulp establishes a network of reticular cells, which accommodate blood vessels and sinuses, and a perifollicular area of lymphocytes around the white pulp (Lametschwandtner et al., 2016). The murine spleen in ALL demonstrates disturbed architecture and especially in T-ALL, white pulp overrules the red pulp and GC organization is compromised (Chen et al., 2015). Even though the *Xenopus* spleen lacks GCs, in leukemic animals tissue architecture is disorganized. CD3 immuno-histochemical staining in tissue sections of spleen is informative to determine abnormalities in the organization of the T cell and B cell zones. Interestingly in thymic sections of leukemic animals in *Xenopus*, the medulla of the thymus invades the cortex and disturbs the architecture of these tissues. This abnormality is obvious in both H&E staining as well as in CD3 stained thymi sections. Furthermore, in murine ALL models, infiltration of liver, spleen and kidneys by lymphoblasts is observed (Jacoby et al., 2014; Pan et al., 2018). Similarly, CD3 immuno-histochemical staining of *Xenopus* liver and kidney tissue sections can easily assess lymphoblasts infiltration. Alternatively, cell suspensions derived from lymphoid organs can be examined for distribution of the same lymphocyte markers that are used in whole blood flow cytometric analysis.

### Genotypic Analysis of F_0_ Crispants (Figure 1)

**FIGURE 2:**
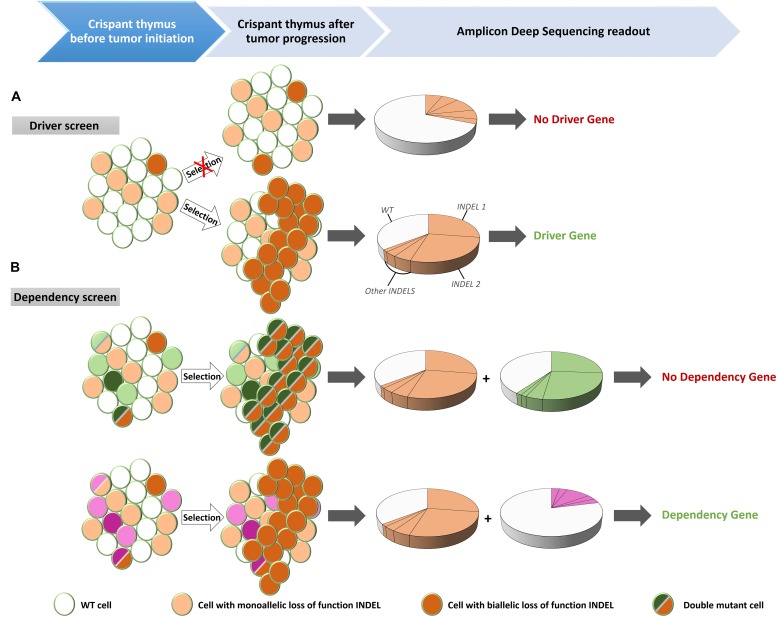
Experimental set-up for the identification of driver mutations in tumor suppressor genes (TSG) and for dependency factors. **(A)** Injection of Cas9 recombinant proteins and guide RNAs targeting putative TSGs created mosaic mutant animals (crispants) that contain cells with mono-allelic or bi-allelic inactivation of the driver genes. Cells with bi-allelic inactivation will have a proliferative advantages over the wild type (WT) or mono-allelic mutant cells. When disease is observed, DNA is extracted from the thymus and after PCR amplification of the targeted regions, the PCR amplicon is subjected to deep sequencing. The presence of two unique frame shifting INDELs at equal proportion is indicative of a clonally expanding subpopulation, characteristic for leukemic disease. **(B)** Multiplexing of guide RNAs targeting a known driver gene together with a candidate dependency factor gene. If the candidate is not a dependency factor, clonal expanding cells will be present – identified by the INDEL signature of the driver gene (orange) – with bi-allelic inactivation of the analyzed gene (green) (top). However, in case of a genuine dependency factor gene (purple), expanding clones will never contain bi-allelic inactivation of this gene (bottom).

Leukemia is a disease of the hematologic system that results in clonal expansion of malignant lymphoblasts. Depending on the stage of oncogenic transformation, the clone of malignant cells can show a uniform rearrangement of their BCR (B-ALL) or TCR (T-ALL; Schultz and Farkas, 1993). This genomic BCR/TCR rearrangement can be used as a signature to identify an expanded malignant clone and can be detected by Southern Blot analysis (Schultz and Farkas, 1993) or by Rapid Amplification of cDNA Ends (RACE; Rosati et al., 2017). We apply alternative methods exploiting the specifics of the genome editing techniques. Mosaic mutant animals generated by CRISPR/Cas9, that bear frame shifting LOF mutations in TSGs have unique genomic features. When Cas9 creates double strand breaks (DSBs) in targeted DNA sequences, the majority of DSBs is repaired by Non-Homologs End Joining (NHEJ). NHEJ repairs the DSBs by incorporating or omitting random nucleotides (Zhang et al., 2014; Wang et al., 2016). As a result, variable patterns of insertions/deletions (INDELS) are generated in the cells of the F_0_ crispants (Naert et al., 2017). However, the leukemic cells of a F_0_ crispant will present with clear enrichment for limited INDEL patterns. Therefore, an effective way to evaluate clonal lymphoblasts expansion is to subject the PCR amplicon of the CRISPR targeted region to Next Generation Sequencing (NGS) (Figure 1). For T-ALL, thymi, which are the main localization site for the T cell lymphoblasts, are dissected from F_0_ crispants and genomic DNA is extracted. The area of interest (i.e., the target site of the sgRNA) in the CRISPR/Cas9 targeted gene(s), is PCR amplified, bar-coded and included in a NGS run. INDEL patterns can subsequently be detected by BATCH-GE analysis (Boel et al., 2016) and evaluated for the presence of dominant clones (Figure 2A).

## Challenges for Modeling Hematologic Malignances in *X. Tropicalis*

Some complications and challenges for modeling hematologic malignancies still remain. *X. tropicalis* has a small size compared to *X. laevis*. Cardiac puncture in tadpoles with glass capillary needles is challenging and only small amounts of blood can be collected. Larval blood cells are also very fragile compared to adult blood. The small blood volumes restrict subsequent phenotypic analysis, especially flow cytometry. Detailed immunophenotyping of the blood from a symptomatic animal is not possible. However, these complications could be partially bypassed by using transgenic lines such as *rag2:EGFP*. Indeed, the *rag2:EGFP* line in zebrafish has greatly facilitated the analysis of the leukemic models in this organisms. This transgenic line allows to gate out lymphocytes from other blood cell types and thus reduces the number of cell markers to be used. In addition, it allows for *in vivo* detection of leukemic animals, facilitates follow up of disease progression, and provides the opportunity for the development of transplantation assays for further disease characterization.

## Future Perspectives and Directions

In addition to current cancer models, *X. tropicalis* could expand the toolbox of model systems for detailed *in vivo* characterization and identification of novel therapeutic strategies. It shares with zebrafish the external development and the associated ease with which to generate mosaic or full knockout models, but has the unique property of a true diploid genome. In addition, although it still lacks some typical secondary lymphoid organs found in mammals, like lymph nodes and GCs, other features such as a clear separation of the red pulp and white pulp in the spleen and canonical class switch recombination of immunoglobulin receptors are still absent in teleosts (Neely et al., 2018). Exploiting the easy and efficient CRISPR/Cas9 mediated multiplexed genome editing and positive clonal selection of leukemic cells will allow the identification of novel driver and modifier mutations (Figure 2A). Alternatively, an existing crispant *Xenopus* leukemic model can be exploited for screening of genes that are essential for proliferation and/or viability of the leukemic cells, the so-called dependency factors (McDonald et al., 2017) (Figure 2B). This application is well established in our lab for solid tumor models (Naert et al., unpublished). This efficient pipeline could be transferred to our ALL model by disrupting the gene of interest with CRISPR/Cas9. Since dependency factor expression is under positive selection, there will be selective pressure to maintain at least one functional allele in the expanding leukemic clones. Besides identifying new target genes for therapies, our model can also be used for compound screening. We have already successfully treated F_0_ crispants exhibiting solid tumors with candidate compounds. The experimental approach was straightforward since compounds could be easily taken up by the animal via the water by several routes (gills, skin, water uptake) or alternatively by intraperitoneal injection. The administration route is determined by the (water) solubility and the price of the compound. Poorly soluble or very expensive compounds are preferentially injected intraperitoneally. Evidently, a critical point is the amount of compound to be administered to the animals. For injections, we rely on the concentrations and injection schemes described for the compound in mouse studies. For administration via the water, we base the concentrations on cell culture studies. Toxicity can first be tested on early pre-feeding tadpoles. We usually start with a five-fold concentration of the IC50 value. Ideally a biological response (e.g., modification of a protein substrate or expression of a known target gene) is used as a readout for effectiveness of the compound (e.g., in lysed liver extracts). Evidently it is important to obtain ethical approval for these treatments.

The previously mentioned use of *rag2* knockout lines for cancer cell grafting may ultimately be not straightforward. Evidently, these animals still have an innate immune system where for instance natural killer cells could still affect the transplanted cells. Also, these animals may be prone to disease. Therefore, an interesting future direction will be the use of *X. tropicalis* inbred strains. This will be a great tool for grafting of cancer cells directly from a diseased donor or from cell lines established derived from these strains. Several inbred strains are currently available from the National BioResource Project (NBRP), the Japanese *X. tropicalis* stock centeraaa.^1^

In sum, *X. tropicalis* is an emerging organism for modeling hematologic malignancies. With some investment, this aquatic vertebrate could become a strong tool for laboratories that are active in identifying new molecular targets for therapy as well as new driver mutations in hematological malignancies.

## Ethics Statement

Approval of the experiments was obtained from the Ethical Committee for Animal Experimentation, Ghent University, Faculty of Sciences.

## Author Contributions

DD and KV wrote the manuscript. DT and PVV provided experimental know-how and input on human leukemia.

## Conflict of Interest Statement

The authors declare that the research was conducted in the absence of any commercial or financial relationships that could be construed as a potential conflict of interest.

## Footnotes

1 http://nbrp-xt.hiroshima-u.ac.jp/xenobiores_en/iweb_en/Top.html
