# Corrigendum: *Xenopus tropicalis*: Joining the Armada in the Fight Against Blood Cancer

**DOI:** 10.3389/fphys.2019.00210

**Published:** 2019-03-14

**Authors:** Dionysia Dimitrakopoulou, Dieter Tulkens, Pieter Van Vlierberghe, Kris Vleminckx

**Affiliations:** ^1^Department of Biomedical Molecular Biology, Ghent University, Ghent, Belgium; ^2^Cancer Research Institute Ghent (CRIG), Ghent, Belgium; ^3^Department of Biomolecular Medicine, Ghent University, Ghent, Belgium

**Keywords:** *Xenopus*, CRISPR/Cas9, leukemia, T-ALL, genome editing, thymus, tumor suppressor genes, cancer

An author name was incorrectly spelled as **“Pieter Van Vlieberghe.”** The correct spelling is **“Pieter Van Vlierberghe.”**

The authors apologize for this error and state that this does not change the scientific conclusions of the article in any way. The original article has been updated.

